# Amniotic fluid embolism: A puzzling and dangerous obstetric problem

**DOI:** 10.1371/journal.pmed.1002976

**Published:** 2019-11-12

**Authors:** Sarka Lisonkova, Michael S. Kramer

**Affiliations:** 1 Department of Obstetrics and Gynaecology, University of British Columbia, Vancouver, British Columbia, Canada; 2 Department of Pediatrics and of Epidemiology, Biostatistics and Occupational Health, McGill University, Montreal, Quebec, Canada

## Abstract

In a Perspective, Sarka Lisonkova and Michael Kramer discuss the accompanying study by Kathryn Fitzpatrick and co-authors on management of amniotic fluid embolism.

Owing to its uncertain etiology, varying symptoms, rapid onset, and high fatality rate, amniotic fluid embolism (AFE) is one of the most challenging obstetric emergencies. In a new international study published in *PLOS Medicine*, Kathryn Fitzpatrick and colleagues [1] provide valuable clinical information about this rare complication, which occurs in 2–8 of 100,000 pregnancies [2]. The clinical signs and symptoms of AFE include a rapid deterioration of maternal condition, cardiac arrest or arrhythmia, hypotension, respiratory distress, coagulopathy and massive hemorrhage, and acute fetal compromise. Premonitory symptoms such as tingling, shortness of breath, and agitation may occur before the signs and symptoms of cardiovascular collapse. Consumptive coagulopathy without cardiorespiratory symptoms is sometimes recognized as a forme fruste of AFE, but it is important to exclude other possible diagnoses, such as septic shock or coagulopathy caused by, rather than the cause of, excessive bleeding. Myocardial infarction and other conditions can also resemble AFE. Given the acuity and complexity of AFE signs and symptoms, an immediate response by a multidisciplinary team including experienced specialists in obstetrics, maternal–fetal medicine, anesthesia, intensive care, and hematology is probably key for survival, as observed by Fitzpatrick and colleagues [1].

Because AFE is a diagnosis of exclusion, a precise case definition is difficult to establish. For these reasons, Fitzpatrick and coauthors examined risk factors, prognosis, and clinical management of AFE using three different definitions: the most liberal definition proposed by the United Kingdom Obstetric Surveillance System (UKOSS) [3], a consensus-driven definition developed by the International Network of Obstetric Survey Systems (INOSS) [4], and the most restrictive definition developed by Clark and colleagues and used by the Amniotic Fluid Embolism Registry in the United States [5]. The latter two definitions were modified to harmonize the data collected across international sites. Interestingly, the main risk factors identified in this study were consistent across all three case definitions. Prenatal risk factors included advanced maternal age, multiple pregnancy, gestational diabetes, polyhydramnios, placenta previa, and placental abruption. Several risk factors were related to common obstetric interventions: induction of labor, operative vaginal delivery, and cesarean delivery. This new evidence corroborates the results of previous studies [2,6–9] and should spawn further etiologic research. Knowledge of these risk factors has little utility for clinical prediction of AFE, however, because the vast majority of women with these risk factors will have a normal pregnancy and delivery.

In previous studies, the case fatality of AFE varied between 11% and 48%, depending on the study design (population versus hospital based) and case definition [2,6]. Using the UKOSS case definition, Fitzpatrick and colleagues observed a case fatality rate of 21%, whereas the INOSS and Clark case definitions yielded case fatality rates of 29% and 24%, respectively. The new study further confirms the unsurprising association between cardiac arrest and poor outcome.

The AFE presentation that dominates the main clinical picture—e.g., coagulopathy (with or without massive bleeding), refractory pulmonary hypertension, or neurological symptoms—should dictate the therapeutic approach. Even before the diagnosis of AFE is established, high-quality cardiopulmonary resuscitation (CPR) should be the first response to cardiac arrest, followed by ongoing cardiorespiratory support. Optimal volume management in response to changing hemodynamics is also crucial, although it is difficult to strike the correct balance between maintaining cardiac output and preventing fluid overload and pulmonary edema.

To some degree, the varying treatment modalities reported by Fitzpatrick and colleagues reflect the variation in presenting symptoms among the AFE cases included in their study. Because most of the cases had coagulopathy and bleeding, coagulation management and surgical interventions to stop the bleeding appeared to improve overall survival. The interventions included the administration of concentrated fibrinogen, platelets, and transfusion of whole blood or red blood cells. All these interventions, however, are contingent upon successful CPR in cases with initial cardiopulmonary arrest, a sine qua non for survival. Even though the authors’ analyses of AFE management modalities were statistically adjusted for cardiac arrest (as a marker of AFE severity), the strength of the protective association with these modalities is highly likely to be biased by reverse causality. Receipt of any intervention depends on survival until the time it is administered, and thus survival may be a cause, rather than a consequence, of the intervention. Nevertheless, Fitzpatrick and colleagues are correct in drawing attention to early detection and correction of coagulation deficiencies in suspected AFE cases, as well as to potentially important newer treatments such as tranexamic acid.

Concerns for fetal well-being add to the gravity of AFE. Acute fetal distress often accompanies the underlying triggers of AFE (e.g., placental abruption) and hypoxia caused by maternal respiratory or cardiac arrest or severe hemorrhage. Expedited delivery is important in women with AFE at 23 weeks’ gestation or later to stabilize the mother and prevent fetal demise. In most severe cases, emergency cesarean delivery should occur within 5–10 minutes after cardiac arrest to prevent fetal or neonatal death, but that is seldom possible. Rates of stillbirth and early neonatal death after AFE are as high as 40% [2,10].

AFE is one of the leading causes of maternal death in industrialized countries, but unanswered questions remain about its etiology, prediction, and optimal management. Multicenter international data with a high level of clinical detail, as reported by Fitzpatrick and colleagues, are essential for further progress. Additionally, a common definition and uniform set of collected data are needed to establish a large pool of AFE cases for future surveillance and research. The “strict definition” proposed by Clark and colleagues [5] is more likely to yield a homogeneous cohort by excluding potentially misdiagnosed (false-positive) cases.

From the clinical standpoint, AFE does not match the typical picture for embolism and more closely resembles conditions such as anaphylaxis or systemic inflammatory response syndrome [10]. Future research should therefore focus on genetic and clinical determinants of maternal susceptibility to such an aberrant response to amniotic fluid or fetal cells. Because a reliable diagnostic test for AFE is not currently available, better biomedical markers and other predictors are also needed to improve preventive and therapeutic interventions. Meanwhile, a multidisciplinary approach to AFE management, coupled with tertiary-level hospital resources, remains the best approach to improving survival of both the mother and her baby.

## Abbreviations

AFE: amniotic fluid embolism
CPR: cardiopulmonary resuscitation
INOSS: International Network of Obstetric Survey Systems
UKOSS: United Kingdom Obstetric Surveillance System
